# Experimental Infection of Pigs with Recent European Porcine Epidemic Diarrhea Viruses

**DOI:** 10.3390/v14122751

**Published:** 2022-12-09

**Authors:** Christina M. Lazov, Louise Lohse, Graham J. Belsham, Thomas Bruun Rasmussen, Anette Bøtner

**Affiliations:** 1 National Veterinary Institute, Technical University of Denmark, 4771 Kalvehave, Denmark; 2 Section of Veterinary Clinical Microbiology, Department of Veterinary and Animal Sciences, University of Copenhagen, 1870 Frederiksberg, Denmark; 3 Section for Veterinary Virology, Department of Virus and Microbiological Special Diagnostics, Statens Serum Institut, 2300 Copenhagen, Denmark

**Keywords:** coronavirus, experimental infection, swine enteric coronavirus (SeCoV), porcine epidemic diarrhea virus (PEDV)

## Abstract

Porcine epidemic diarrhea virus (PEDV), belonging to the genus *Alphacoronavirus*, can cause serious disease in pigs of all ages, especially in suckling pigs. Differences in virulence have been observed between various strains of this virus. In this study, four pigs were inoculated with PEDV from Germany (intestine/intestinal content collected from pigs in 2016) and four pigs with PEDV from Italy (intestine/intestinal material collected from pigs in 2016). The pigs were re-inoculated with the same virus on multiple occasions to create a more robust infection and enhance the antibody responses. The clinical signs and pathological changes observed were generally mild. Two distinct peaks of virus excretion were seen in the group of pigs inoculated with the PEDV from Germany, while only one strong peak was seen for the group of pigs that received the virus from Italy. Seroconversion was seen by days 18 and 10 post-inoculation with PEDV in all surviving pigs from the groups that received the inoculums from Germany and Italy, respectively. Attempts to infect pigs with a swine enteric coronavirus (SeCoV) from Slovakia were unsuccessful, and no signs of infection were observed in the inoculated animals.

## 1. Introduction

Porcine epidemic diarrhea (PED) is a disease caused by a porcine coronavirus (termed PEDV) classified within the *Alphacoronavirus* genus [1]. Other porcine alphacoronaviruses include transmissible gastroenteritis virus (TGEV) and its deletion variant, porcine respiratory coronavirus (PRCV) [2]. Fecal-oral, fecal-nasal, or indirect transmission of PEDV between pigs of all ages results in acute enteritis, exfoliation, malabsorptive and maldigestive diarrhea, as well as vomiting and decreased appetite [3].

PEDV was first observed in Europe in the 1970s, but its occurrence gradually declined in the 1980s. However, a few disease outbreaks were seen sporadically, e.g., in Belgium in 1992, in England in 1998, and in Italy from 2005 to 2006 [4]. During this period, PEDV spread to Thailand, Korea, and China; the virus became endemic in many regions of Asia, significantly impacting the pig industry [5]. In 2013, the USA, which had previously been free from the disease, experienced outbreaks of PED, resulting in up to 95% mortality in suckling pigs [6]. About 7 million piglets died there within a year due to this disease [7]. The epidemic of PED that swept across the Americas in this period included two different PEDV variants, described as non-INDEL and INDEL strains, sometimes together with a newly discovered porcine deltacoronavirus [8]. The presence of certain insertions characterized the PEDV INDEL variants and deletions in the spike (S) protein gene, compared to the non-INDEL strain [9,10]; the S protein is required for the interaction of the virus with the cellular receptor and for inducing neutralizing antibodies [11]. The identity of the cellular receptor for PEDV is not yet established, but it was recently suggested to be pDC-SIGN (porcine dendritic cell-specific intercellular adhesion molecular-3 capture non-integrin) [12]. In 2014, PEDV strains closely related to the INDEL strains in the USA spread to Europe. These resulted in outbreaks in several countries, but not in the same epidemic fashion as in the USA. However, most of the pig population in Europe had a similar naïve immune status [4,13].

Following the introduction of the INDEL variant into Europe, the number of samples submitted for diagnostic testing initially rose but has now dropped again. However, the presence of PEDV still causes clinical disease on farms, and the strains continue to evolve. Most changes have been detected in the coding sequence for the S1 (N-terminal) region of the S protein [14]. In some European countries, a few outbreaks of viral diarrhea initially attributed to PEDV have now been shown to have been caused by the recombinant swine enteric coronavirus (SeCoV) with a genetic backbone of TGEV/PRCV but including the S protein gene from PEDV [15,16,17,18]. Although the S gene in SeCoV resembles that of PEDV, the precise origins of these recombinant viruses have not yet been identified, as phylogenetic analysis of the S gene sequence separates SeCoV from known PEDVs [18].

Previously, we have described experimental infections of pigs with a non-INDEL PEDV from the USA and with an older European cell-culture-adapted strain (Br1/87) [19]. In those previous studies, we obtained an isolate of the US strain in cell culture from the intestinal contents of the inoculated pigs. This new study aimed to characterize the properties of porcine coronaviruses recently circulating in member states of the European Union under defined experimental conditions and to observe clinical signs, describe pathological lesions, and follow the immune response. In addition, we aimed to generate infectious organ material for virus isolation and produce antisera in the infected pigs.

## 2. Materials and Methods

All experimental procedures, animal care, and maintenance were conducted in accordance with Danish and EU legislation (Consolidation Act 474 15/05/2014 and EU Directive 2010/63/EU). Water was provided ad libitum, and the pigs were fed a commercial diet for weaned pigs once a day. Straw was used for bedding.

### 2.1. Animals and Housing

In total, eight pigs, 28 days old, were transferred to the high containment facility at the National Veterinary Institute, Lindholm. Two individual isolation units were used to house the two groups of animals, with four pigs in each (pigs 1–4 in group 1 and 5–8 in group 2). The pigs were obtained from a commercial farrow-to-finish farm with specific pathogen-free (SPF) status, including freedom from the pathogens: porcine reproductive and respiratory syndrome virus (PRRSV), *Mycoplasma hyopneumonia*, *Actinobacillus pleuropneumonia*, *Brachyspira hyodysenteria*, *Pasteurella multocida*, scabies, and lice. All pigs were found to be healthy by veterinary inspection upon arrival, four days before the start of the experiment, which was designated as post-infection day 0 (PID 0).

### 2.2. Virus Inoculation

Inoculation was performed orally using a syringe with 2–4 mL of virus suspension, i.e., 10% fecal suspension and/or intestinal homogenate in Eagle’s minimal essential medium (EMEM). Briefly, group 1 was inoculated with PEDV containing fecal material from Germany (kindly provided by S. Blome, FLI) from an experimental study as described previously [20], and group 2 with SeCoV in samples from Slovakia [16,21] (see Table 1). Additional inoculations with different virus preparations were performed on PID 11, 24, and 39. For group 1, the inoculum again contained the PED virus of German origin through the use of material from pig 1. In contrast, group 2 was inoculated with PEDV material originating from diarrheic piglets in Northern Italy in May 2016 [22]. All inoculation materials are described in more detail in Table 1.

### 2.3. Sampling, Sample Analysis, and Scoring of Pigs

The pigs were clinically assessed daily; seven parameters reflecting the overall condition and gastrointestinal status of each pig were scored from 0 (normal) to 1, 2, or 3 (most severe), totaling a maximum score of 12 per pig per day as previously described [19]. Rectal temperatures were measured daily until PID 25, and fecal swabs, or fecal samples, were collected daily in the first week and with decreasing frequency from the second week. Nucleic acids were extracted from the fecal swabs or 10% suspensions of fecal samples in EMEM using the MagNaPure 96 robot with the DNA and Viral NA Small Volume kit (Roche Diagnostics) and eluted in nuclease-free water (50 μL). For detection of PEDV, real-time RT-qPCRs, with 40 cycles, were performed on the Stratagene Mx3005P (Agilent Technologies, Glostrup, Denmark) using the One-Step real-time RT-PCR kit (Qiagen) with probe and primers targeting the PEDV nucleocapsid (N) gene [23,24] as described previously [19]. One reverse primer and two forward primers and probes (with the same fluorophore) matching either the early EU strains or the newer US strains were used simultaneously. Primers targeting the TGEV/PRCV N gene and a slightly modified probe containing the sequence 5′-TGGCACTGCTCCCATTGGCAACGA-3′ [24] were mixed with the Applied Biosystems™ RNA UltraSense™ One-Step Quantitative RT-PCR System (Thermo Fisher Scientific, Waltham, MA, USA) kit and used similarly for monitoring the presence of viral RNA from SeCoV. Blood samples (5 mL) for serological analysis were collected on PID 0, 4, 7, 10, 15, 18, 21, 24, 32, 39, 49, and 52. The sera were analyzed for the presence of anti-PEDV antibodies using an “in-house” PEDV blocking ELISA and with an immunoperoxidase monolayer assay (IPMA) for serum titration, performed as previously described [19,25]. Essentially, in the blocking ELISA, whole virus particles of PEDV strain Br1/87 were used to coat ELISA plates overnight at 4 °C. Test sera (1:10 and, if necessary, a 4-fold dilution) were added to the plates and incubated overnight at 4 °C. The presence of antibodies to PEDV block, or partially block, epitopes recognized by an added biotinylated anti-PEDV polyclonal antibody (1:100 dilution, in-house production) that was added for 1 h and incubated at room temperature. The bound biotinylated anti-PEDV antibodies were detected using eBioscience™ peroxidase-labeled avidin (1:15,000, Thermo Fisher Scientific) plus substrate (3,3′,5,5′-tetramethylbenzidine (TMB-One), Thermo Fisher Scientific). Incubation was for 15 min at room temperature prior to the addition of a stop solution (0.5 M sulfuric acid). Additionally, a commercial blocking ELISA (Svanovir TGEV/PRCV-Ab assay, Svanova) was used to detect antibodies against TGEV/PRCV in serum samples from group 2 pigs. The absorbance of the ELISA wells was measured at wavelengths of 450/620 nm using the ELx808™ ELISA plate reader (Agilent Technologies). The readings were used to calculate the absorbance percentage (ODp) values using the formula: ODp = (sample OD × 100/(mean OD NIR) where NIR = non-inhibited reference serum (4 wells were used per plate to determine a mean value for this). In the IPMA, PEDV-infected Vero cells (using 100 TCID_50_ per well) were fixed and permeabilized with ice-cold ethanol (99.9%). The intracellular PEDV proteins were visualized by incubation with (in turn): anti-PEDV polyclonal antibody (using test sera or a known positive control (in-house production), using 1:10 and 4-fold dilutions, and incubation for 1 h at 37 °C), a rabbit anti-swine antibody conjugated with horseradish peroxidase (1:200 dilution, Dako, Agilent Technologies, incubation for 30 min at 37 °C) and the substrate 3-amino-9-ethyl carbazol (Sigma-Aldrich, St. Louis, MO, USA) (0.04% in ethanol (10%)) with hydrogen peroxide (2.5 µL of 30% solution added per 5 mL of the substrate); incubation was for 20 min at room temperature. Red-stained cells were scored using a microscope. Antibody titers were calculated as the reciprocal of the highest dilution of the test serum that gave a positive result in the IPMA.

### 2.4. Euthanasia and Necropsies

The pigs were euthanized for necropsy at different time points post-infection according to when pathological lesions were expected during peaks of virus excretion (at PID 4 and PID 15). The surviving pigs were kept until after seroconversion (PID 52). The animals were sedated with Zoletil^®^ (Virbac), and a maximum of 140 mL of blood was collected. They were then euthanized using intravenous injection of sodium pentobarbital (SCANVET) and exsanguinated. The four pigs euthanized on PID 4 and PID 15 were subjected to necropsy, and selected organs were sampled.

### 2.5. Virus Isolation

Following the necropsies, attempts were made to isolate the virus from the intestinal material of the four pigs euthanized on PID 4 and PID 15. Intestinal contents and jejunum and ileum intestinal linings were harvested by scraping and suspended (1:10 by volume) in EMEM with antibiotics (mix of streptocillin, neomycin, amphotericin, and benzylpenicillin). The suspensions were centrifuged for 10 min at 3000 rpm, and the supernatants passed through 0.45 µm filters. Cells used for inoculation were seeded in 6-well culture plates or 10 mL flasks. The preferred cell line for the virus isolation attempts was Vero cells (ATCC, CCL-81), but also MARC-145 cells (kindly provided by Dr. Ferrari, IZSLER, Brescia, Italy), IB-RS-2 D10 cells (ATCC, CRL-1835) and ST cells (ATCC, CRL-1746) were used. The Vero cells were grown in EMEM supplemented with 0.3% tryptose broth and 5% fetal calf serum (FCS). About one hour before inoculation, the cell monolayer was rinsed and supplied with fresh virus growth medium, which contained EMEM (without FCS) with tryptose (0.3%) plus further added yeast extract (0.02%) and trypsin (10 µg/mL) for virus inoculation and growth. Virus isolation procedures, with variable setups, were performed, including sample dilution in virus growth medium (1:5, 1:10, 1:20, 1:50), CO_2_-enriched environment (5%), gentle rocking or fixed position for incubation, the addition of 5% FCS in the virus growth medium from two hours post inoculation and also the absence of trypsin prior to inoculation. The cells were microscopically assessed for cytopathic effect (CPE) on a daily basis and compared to control cells. When CPE was observed, or after a maximum of three days of incubation, the cells were frozen at −80 °C. New virus passages were made by inoculating fresh cells with supernatants from thawed and centrifuged cell harvests. Assessment of virus growth in individual cell harvests was performed by RT-qPCR analysis of nucleic acids extracted from undiluted cell supernatant or by fixation and staining of inoculated cells using the IPMA, as above.

## 3. Results

### 3.1. Inoculation of Pigs and Analysis of Infection

**Figure 1:**
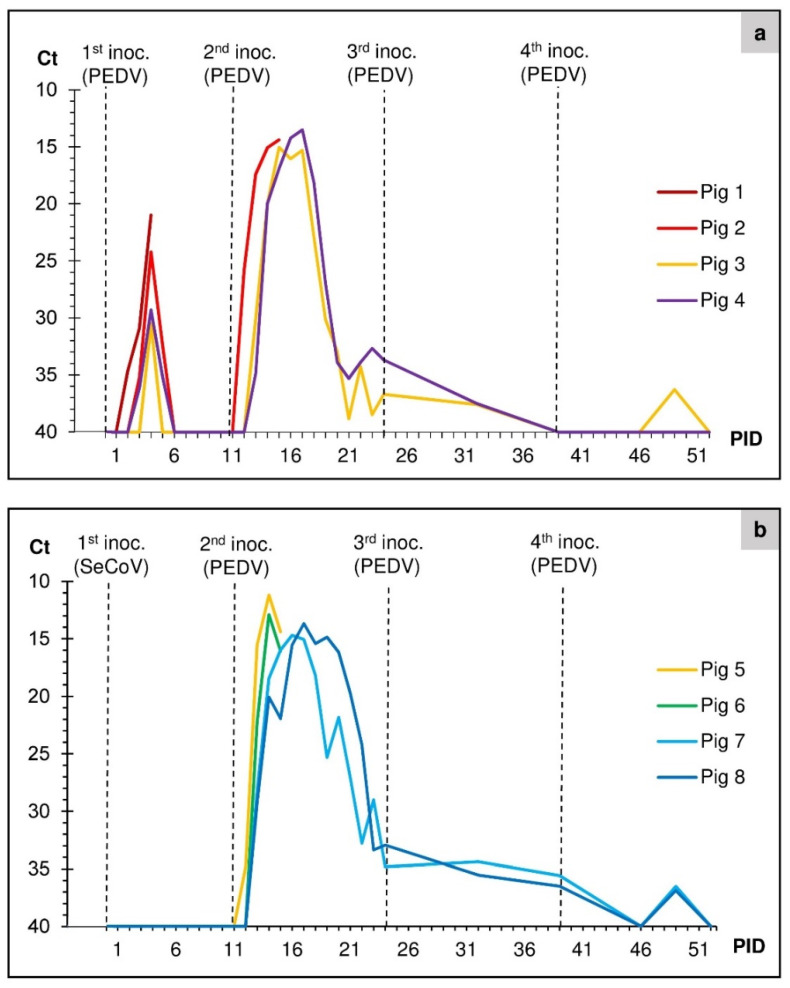
Excretion of PEDV in fecal samples as determined by detecting PEDV RNA using RT-qPCR. Tested samples were given the value of 40 if the cycle threshold (Ct) was not reached after 40 cycles. Panel (**a**) shows the results from pigs in group 1 inoculated with PEDV material from Germany with the 1st–4th inoculations (inoc.) on PID 0, 11, 24, and 39. Pig 1 was euthanized on PID 4, and pig 2 on PID 15. Panel (**b**) shows the results from pigs in group 2 initially inoculated with SeCoV on PID 0 and subsequently with PEDV from Italy on PID 11, 24, and 39. Pigs 5 and 6 were euthanized on PID 15.

Pigs in group 1 were initially inoculated orally with a suspension of fecal material containing PEDV from Germany. During the following days, the pigs showed only mild clinical signs and virus excretion (as measured by RT-qPCR) that subsided after a few days (Figure 1a). Pig 1 was euthanized at PID 5 and necropsied. The surviving three pigs in group 1 were re-inoculated with PEDV on PID 11, this time using material collected from the euthanized pig 1, resulting in a more robust infection (see Figure 1a).

Pigs in group 2 were initially inoculated with fecal/intestinal homogenate containing SeCoV obtained from Slovakia. There was no detectable virus excretion in any of the four pigs in the days following this inoculation (Figure 1b), and no signs of disease were apparent. Therefore, it was decided to inoculate all of the pigs for a second time on PID 11. However, on this occasion, the pigs were inoculated with fecal material containing PEDV obtained from Italy, as described by Boniotti et al. [22]. This resulted in high levels of PEDV excretion (Figure 1b).

In order to obtain robust antibody responses in the pigs, two further inoculations were performed on PID 24 and 39 using the same material as used on PID 11 for both groups. The serum from all the pigs was harvested at the end of the experiment on PID 52 for scientific and diagnostic purposes.

### 3.2. Presence of Viral RNA Measured by RT-qPCR

Primary inoculation materials and fecal swabs from all eight pigs were tested prior to the first inoculation for the presence of PEDV and TGEV/PRCV using RT-qPCR. The presence of PEDV RNA observed by testing daily fecal swabs and fecal samples are shown in Figure 1. In group 1, in the pigs inoculated with the PEDV from Germany (Figure 1a), virus excretion started on PID 2 (pig 1) or PID 3 (pigs 2–4), and viral RNA levels peaked on PID 4. Still, only rather low levels of virus excretion were detected (the highest excretion was seen in pig 1, Ct value of ca. 21). After the second inoculation on PID 11 with material harvested from pig 1, another, much higher peak of viral RNA excretion was seen, the Ct values became as low as 13.5 for pig 4 on PID 17, indicating that the virus excretion increased >100-fold from the first peak. No further peaks of virus excretion were seen for the pigs in group 1 following subsequent re-inoculations (Figure 1a), but a low level of viral RNA was still detectable in fecal samples from pig 3 at PID 49.

The pigs in group 2 (Figure 1b) were initially inoculated with material containing SeCoV. Assays for the SeCoV RNA in this inoculation material gave relatively high Ct values (from 25–35, using the TGEV/PRCV N gene assay, indicative of relatively low levels of virus) and each of the 4 inoculated pigs in group 2 tested negative in this assay in all fecal samples and swab samples collected between PID 0 and PID 11. Thus, there was no sign of active infection with this virus. Therefore, it was decided to switch to using PEDV material from Italy for a second inoculation. Following this inoculation, PEDV excretion was first detected in pig 5 on PID 12, just one day after the first PEDV inoculation. The virus excretion peaked on PID 15 for pigs 5 and 6, on PID 17 for pig 7, and on PID 18 for pig 8—i.e., three to six days after inoculation (with Ct values between 11.1 and 14.7). No further peaks of virus excretion were observed for animals in group 2 following the repeat inoculations on PID 24 and 39 (see Figure 1b). Still, PEDV RNA was detected intermittently in the fecal samples until PID 49 (38 days post-first PEDV inoculation).

### 3.3. Antibodies Measured by ELISAs and IPMA

**Figure 2:**
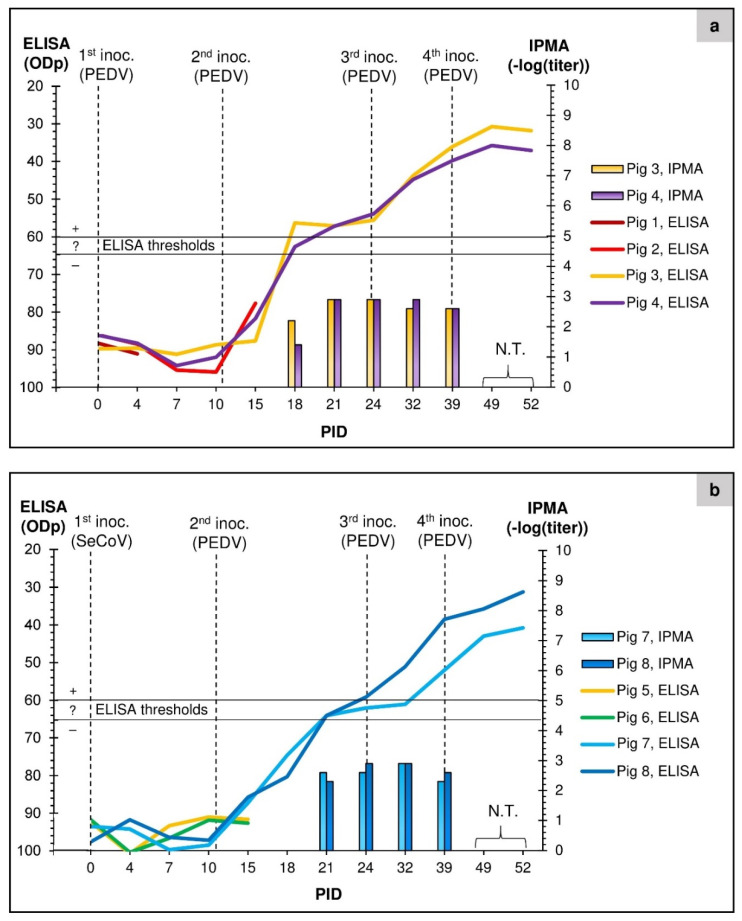
Seroconversion of pigs in group 1 (panel (**a**)) and group 2 (panel (**b**)) against PEDV. The average ELISA absorbance percentage (ODp) values from testing of serum samples collected on the pre-selected days indicated on the x-axis are shown. The ELISA threshold range of ODp values from 65 to 60 was defined as inconclusive, and values below 60 were considered positive. The columns show the negative log_10_ titers, defined as the highest dilution of serum that gave a positive result in the IPMA. The IPMA was performed on sera from all the indicated days except PID 49 and 52 (not tested, N.T.).

Prior to inoculation, the eight pigs all tested negative for antibodies against PEDV and TGEV using ELISA. Measurements of antibodies specific for PEDV detected using the “in-house” PEDV blocking ELISA and IPMA are shown in Figure 2a,b. No anti-PEDV response was apparent in the pigs in group 1 prior to the 2nd inoculation (on PID 11). However, in both the ELISA and IPMA, seroconversion against PEDV in these pigs was detected from PID 18 (7 days after the re-inoculation) (Figure 2a). Thus, the 3rd and 4th inoculations were performed in seropositive animals, and this may account for the lack of further episodes of high virus excretion (Figure 1a).

The pigs in group 2 were tested for antibodies to both TGEV and PEDV before and after inoculation. All tests for TGEV were negative. In these pigs (Figure 2b), the development of antibodies towards PEDV could be detected in both surviving pigs on PID 21 (ten days after the first inoculation with the PEDV from Italy) using IPMA. Using the ELISA, pig 8 scored positive from PID 24 and pig 7 from PID 39, although both pigs reached the inconclusive threshold range for the assay on PID 21. Pigs from group 1 (pigs 1 and 2) and from group 2 (pigs 5 and 6) were euthanized before seroconversion occurred. The antibody responses in both groups (as determined by ELISA) appeared to rise following each PEDV inoculation (Figure 2a,b). However, the titers measured by IPMA seemed to plateau earlier (Figure 2a,b). Presumably, the presence of anti-PEDV antibodies in both groups of pigs by the stage of the 3rd inoculation was sufficient to severely limit further virus replication and excretion.

### 3.4. Clinical Assessment and Necropsies

**Figure 3:**
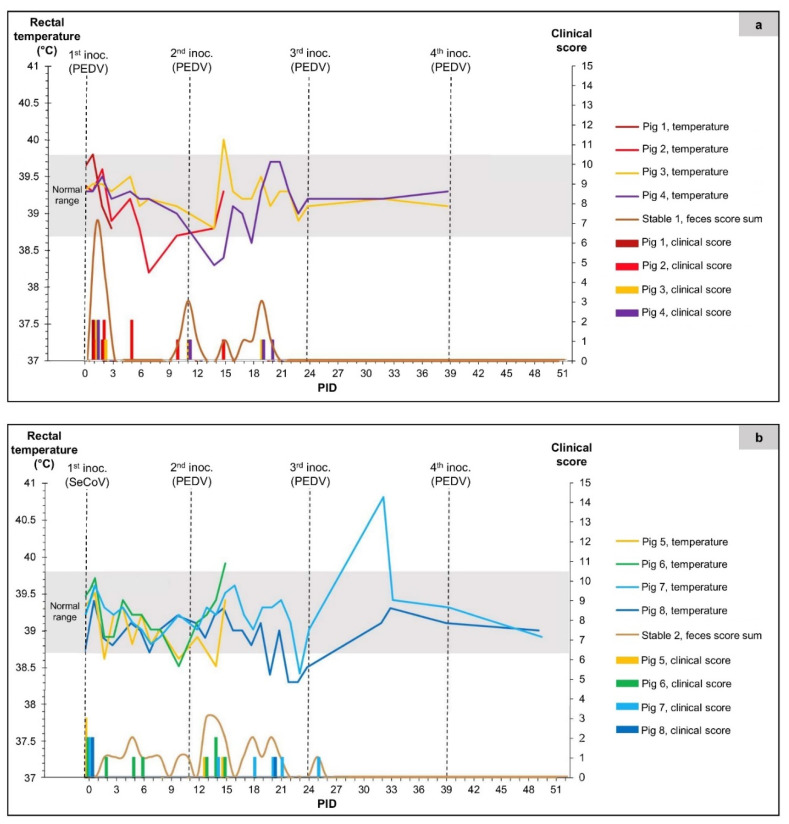
Clinical measurements of the pigs in group 1 (panel (**a**)) and group 2 (panel (**b**)). The sharp curves show the rectal temperature measurements, which were rarely measured near the end of the experiment. The grey bars indicate the normal temperature range for pigs (38.7–39.8 °C). The columns show the clinical scores of the individual pigs in each group. The smooth curve shows the sum of feces scores from the individual pigs, including a score of 1 if thin/non-formed feces or diarrhea were found in the stable.

Overall, only a few clinical signs were observed in the two groups of pigs following inoculation with PEDV and mainly as intermittent diarrhea (clinical score 2) or thin/non-formed feces (clinical score 1) (Figure 3). In group 1 (Figure 3a), after the first inoculation on PID 0, all pigs developed diarrhea from PID 1 or 2 for a duration of 1–2 days. Gastrointestinal signs in the form of thin feces were observed again in the pen around the time of the second inoculation on PID 11 and for up to ten days afterward. A single animal (pig 2) was anorexic (clinical score 2) on PID 5. Still, no other changes in behavior/appearance were observed within this group. Rectal temperatures were mainly within the normal range (38.7–39.8 °C) [26] throughout the experiment, with a single measurement outside of this range for pig 3 on PID 15 (40.0 °C).

Two pigs (1 and 2) from group 1 were subjected to early euthanasia and necropsy on PID 4 (after the initial inoculation) and PID 15 (4 days following the second inoculation on PID 11), respectively. The observations made during necropsies are summarized in Table 2, and RT-qPCR Ct values are given for the intestinal contents or fecal swabs for the sampled days. As indicated in Table 2, pig 1 did not have any internal lesions on PID 4. However, pig 2, euthanized on PID 15, showed mild macroscopic changes with increased fluid in parts of the small intestinal lumen as signs of PEDV infection. All pigs in group 2 had reduced appetite on PID 0. Therefore, the clinical scores were already different from zero before inoculation with SeCoV (Figure 3b). Thin or non-formed feces were observed intermittently from individual pigs and in the stable from PID 2 until PID 25. The only case of diarrhea in this group was seen in pig 6 on PID 14 (three days after the first inoculation with PEDV). Rectal temperatures for the pigs in group 2 were also typically within the normal range, with single measurements outside of the normal range for pig 6 on PID 15 (39.9 °C) and pig 7 on PID 32 (40.8 °C). Two pigs (5 and 6) were subjected to early euthanasia and necropsy on PID 15. They showed mild-to-moderate changes from normal with a general increase of fluid in the intestines (both pigs) together with dilation and gas (pig 6) (Table 2).

### 3.5. Virus Isolation Attempts

Attempts to isolate PEDV from the infected pigs in both group 1 and group 2 were unsuccessful. No virus growth in cell culture was detected by IPMA or by RT-qPCR assays following single or repeated passages. Indeed, it was observed that the Ct values increased after subsequent virus passages (corresponding to dilution and decay of the nucleic acid).

## 4. Discussion

In this study, we wanted to infect young pigs, ca. 4 weeks of age, with recently circulating strains of porcine coronaviruses, i.e., PEDV and SeCoV from Europe to observe and characterize clinical and gross pathological changes due to these infections, to follow the immune responses and to produce clinical materials containing high levels of virus and virus-specific antisera.

Unfortunately, the inoculation of pigs with SeCoV did not result in any signs of infection, and no virus excretion occurred. The inoculation material had readily detectable levels of SeCoV RNA, although these were not high (Table 1), and it is not known whether this RNA was present within infectious virus particles. It could be that the viability of the virus in the material had been compromised before inoculation. In the case description [21], based on the farm outbreak in Slovakia, the infection affected all pigs on the farm, with mortality in young piglets of around 30–35%. The older animals showed loss of appetite, elevated temperatures, and developed diarrhea but without vomiting and with no increase in mortality. Since the outbreak on the two farms in 2015 [16,21], SeCoV has not been detected in other farms in Slovakia. To our knowledge, it is the first time that this recombinant virus has been administered to pigs under experimental conditions. Although other SeCoV strains have been described [15,17,18], it can be difficult to obtain fresh virus-containing material from diseased pigs, as these cases are usually initially attributed to PEDV and only later correctly diagnosed using archived samples.

In our previous study, we were unable to infect pigs with a recent European field strain of PEDV from Germany [19]. Therefore, new material from Germany from an experimental study was obtained, and the pigs in group 1 were inoculated with this. Interestingly, the first inoculation only produced a mild infection in the pigs with low levels of virus excretion, and no antibody responses were apparent at PID 10 (Figure 2a). Hence, it was decided to re-inoculate the group to produce a more robust infection and thorough immunization. Intestinal contents and organ material from the small intestine of pig 1 were used to inoculate the remaining pigs in group 1. Following the 2nd inoculation, the pigs showed a much higher level of virus excretion that continued for the remainder of the experiment (until PID 52). During the necropsy of pig 2 on PID 15, gastrointestinal changes were observed consistent with signs of a mild PEDV infection. Furthermore, seroconversion of the pigs was detected from PID 18. This was a slower response than the seroconversion by PID 7–10 observed in our previous study using a similar experimental setup but with different PEDV strains (US and Br1/87) [19]. However, the anti-PEDV response was detected at 7 days following the 2nd inoculation of the pigs in group 1, which generated a much higher level of virus excretion (see Figure 1a and Figure 2a). A recent review about PEDV reported seroconversion occurring between 7–14 days after primary infection (Jung et al., 2020) [3]. It appeared that the induction of anti-PEDV antibodies was sufficient to severely limit further virus replication and, hence, excretion since no increases in virus excretion were observed in the seropositive animals following re-inoculation on PID 24 and 39 (see Figure 1 and Figure 2). It appears that before seroconversion, there was a time window when it was still possible for PEDV to infect the enterocytes. Considering that the material used for the second inoculation on PID 11 had a Ct value of around 21 (Table 1) and was from a recently euthanized pig (pig 1), it seems that this material contained a substantial amount of viable virus particles that could infect more enterocytes and thereby generate a second, much higher, level of virus excretion than seen after the first inoculation.

Instead of continuing studies with the SeCoV, the uninfected pigs in group 2 were inoculated with PEDV-containing material from a farm in Italy. This inoculation resulted in PEDV infection, and to ensure a robust infection and a strong immune response, this group was re-inoculated repeatedly. The PEDV excretion peaked at 3–6 days after the initial PEDV inoculation and then decreased but continued to be present throughout the experiment (to PID 52, i.e., 41 days post-PEDV inoculation). On PID 13 (2 days after PEDV inoculation), thin feces were found in the pen, and pigs 5 and 6 were euthanized on PID 15. Both pigs showed similar, mild-to-moderate pathological gastrointestinal changes upon necropsy, comparable to pig 2, infected with the PEDV from Germany. The remaining pigs in group 2 seroconverted by PID 21 (IPMA) or PID 24–39 (ELISA), which corresponded to 10 and 13–28 days after the first inoculation with PEDV.

The observed course of the experimental infections in the two groups with PEDV is consistent with reports of recent PEDV outbreaks in Europe, generally described as mild cases [27]. Another factor that probably contributed to the mild disease is the age of the pigs used in the experiment. The pigs in group 1 were 4.5 weeks old at the time of the first inoculation, and the pigs in group 2 were eleven days older by the time they received the PEDV. Normally, PEDV infection shows the most severe disease, with high mortality, in newborn or suckling pigs due to a less developed large intestine and slower regeneration of enterocytes [3]. However, different strains and outbreaks show varying patterns of morbidity and mortality. In an experimental study by Leidenberger et al. [20] that generated the material used in the present study, both inoculated and naturally infected sows showed serious disease with diarrhea, anorexia, and vomiting. Furthermore, suckling piglets without maternally derived antibodies to PEDV were severely affected by diarrhea and vomiting. However, the fattening pigs, which had been the animals mainly affected in the field outbreaks in South West Germany, had generally shown mild clinical signs [13,20]. In Italy, PEDV INDEL strains were responsible for an epidemic wave between 2015 and 2017. In a study evaluating these outbreaks in Northern Italy (mainly in the Po valley), animals in all age groups were affected by diarrhea, but in general, with low mortality rates (highest for piglets) [22]. The material used for inoculation of the pigs in group 2 in the present study was collected from the same geographical region. Sequence analysis of the PEDVs circulating in Germany and Italy at the time that these samples were collected has shown that the viruses were very closely related (>99% identity) to the OH851 S-INDEL variant strain (GenBank accession number KJ399978) from the USA that had been found in 2014 [13,22].

The pigs were RT-qPCR positive for PEDV with intermittent shedding up to PID 49 (for group 2 corresponding to 38 days after PEDV infection). However, we do not know if the long shedding period was due to the re-inoculations and if the third and fourth inoculations had any effect on the duration of virus shedding. Long periods of viral RNA shedding have been described earlier with RT-qPCR detection of the virus for up to 42 days after infection but with the shedding of infectious virus for only 14–16 days [28]. Even longer durations of positive RT-qPCR results, for up to ten weeks after infection of pigs in a field setting, were seen from tests of oral fluid samples [29].

Unfortunately, we were unable to isolate the PEDVs from Germany and Italy, even after in vivo passage in pigs. However, the same methodology had previously been used successfully for isolating a recent PEDV from the USA (US/Lindholm 2014) [19]. In a study in which nine US PEDV strains were successfully isolated, intestines or feces were collected and prepared directly for cell inoculation without freezing and thawing [30]. The authors chose the Vero-81 cell line for this work due to their lower trypsin sensitivity compared to two other Vero cell lines. The inoculated cells were left to incubate for up to seven days to reach about 90% CPE. Challenges in isolating PEDV are well known. The key to success has been suggested to be a high concentration of virus in the inoculum and immediate virus isolation from fresh samples [30,31]. However, isolates of different current European strains of PEDV have been obtained, e.g., INDEL strain isolates from Germany [13]. These isolates are important, as they provide the basis for further characterization of the viruses in the laboratory, in their hosts, and for future vaccine development.

## Figures and Tables

**Table 1:** Overview of inoculation materials used in the infection study.

Inoculation	Group 1 (Pigs 1–4)	Group 2 (Pigs 5–8)
1st on PID 0 (4 pigs per group)	PEDV (Germany): pool of material 6150-D15 and -D16 * (Ct = 20.3)	SeCoV (Slovakia): feces/intestinal homogenate. For pigs 5 and 6 (Ct = 25) and for pigs 7 and 8 (Ct = 30–35)
2nd on PID 11 (3 pigs in group 1, 4 pigs in group 2)	PEDV (Germany): material from pig 1 (Ileum and intestinal contents, Ct = 20.9)	PEDV (Italy): pool of material (Ct = 13–17)
3rd on PID 24 (2 pigs per group)	PEDV (Germany): material from pig 1 (Ileum and intestinal contents)	PEDV (Italy): material from pig 5 (small intestine and intestinal contents, Ct = 14.4)
4th on PID 39 (2 pigs per group)	PEDV (Germany): material from pig 1 (Ileum and intestinal contents)	PEDV (Italy): material from pig 5 (small intestine and intestinal contents)

*: as described previously [20].

**Table 2:** Macroscopic findings during necropsy plus PEDV RNA detection within feces or intestinal contents.

	Group 1		Group 2	
	Pig 1 († PID 4)	Pig 2 († PID 15)	Pig 5 († PID 15)	Pig 6 († PID 15)
External observations	No remarks	Slightly smudged/dirty hindquarters	Normal to lean body condition score	Smudged/dirty hindquarters
Internal findings	No remarks	Accumulation of fluid in some parts of the lumen and slight hyperemia of the small intestine	Accumulation of fluid in the lumen of the small intestine. Watery contents in the ileum. Liquid contents and gas in the colon.	Substantial accumulation of fluid in the lumen and dilation of the small intestine. Liquid contents and some gas in the colon.
PEDV RT-qPCR	Ct 20.9	Ct 14.3	Ct 14.4	Ct 16.0

Note: only abnormal findings are described. † indicates day of euthanisia

## Data Availability

The relevant data from the experiments described here are included in the article.
